# Unpacking Loss to Follow-Up Among HIV-Infected Women Initiated on Option B+ In Northern Tanzania: A Retrospective Chart Review

**DOI:** 10.24248/EAHRJ-D-18-00025

**Published:** 2019-07-30

**Authors:** Festo Mazuguni, Boaz Mwaikugile, Cody Cichowitz, Melissa H Watt, Amasha Mwanamsangu, Blandina T Mmbaga, Prosper Njau, Michael J Mahande, Jim Todd, Jenny Renju

**Affiliations:** a Department of Epidemiology & Biostatistics, Institute of Public Health, Kilimanjaro Christian Medical College, Moshi, Tanzania; b Ifakara Health Institute, Dar es Salaam, Tanzania; c Department of Health, Rombo District Council, Kilimanjaro, Tanzania; d Duke Global Health Institute, Duke University, Durham, NC, USA; e School of Medicine, Johns Hopkins University, Baltimore, MD, USA; f Paediatric and Child Health Department, Kilimanjaro Clinical Research Institute, Moshi, Tanzania; g Prevention of Mother-to-Child HIV Transmission Programme, Ministry of Health, Community Development, Gender, Elderly, and Children, Dar es Salaam, Tanzania; h Department of Population Health, London School of Hygiene & Tropical Medicine, London, UK

## Abstract

**Background:**

In 2014, Tanzania adopted the Option B+ policy for the prevention of mother-to-child transmission of HIV (PMTCT), which stipulates lifelong antiretroviral therapy (ART) for HIV-infected pregnant, postpartum and breastfeeding women, irrespective of CD4 count or WHO clinical staging. Loss to follow-up (LTFU) during pregnancy and the postpartum period may undermine the effectiveness of Option B+. Factors associated with no follow-up (NFU) care, may differ from those associated with LTFU at later time points. This study aimed to identify factors associated with NFU and LTFU among women who initiate ART under Option B+ in Moshi, Tanzania.

**Methods:**

We conducted a retrospective chart review of patients initiating ART on Option B+ between February 2014 and December 2015 in Moshi Municipality, Tanzania. Multivariable log-binomial regression was used to analyse factors associated with NFU. Kaplan-Meier survival functions were used to estimate time to LTFU. Multivariable Cox proportion hazards regression models were used to evaluate variables associated with time to LTFU.

**Results:**

Among 468 women initiating ART under the option B+ programme, 109 (23.3%) had NFU after the initial appointment. Factors associated with increased risk of NFU were: age < 25 years (adjusted hazard ratio [aRR] 1.7; 95% CI, 1.2 to 2.3), initiating ART at a hospital compared to a lower level health facilities (aRR 2.9; 95% CI, 2.1 to 3.9), and having no treatment supporter (aRR 1.5; 95% CI, 1.1 to 2.1). LTFU was higher in women aged < 25 years (aHR 1.4; 95% CI, 1.1 to 1.9), and in women with no treatment supporter (aHR 1.8; 95% CI, 1.4 to 2.3). In women who returned to the clinic after ART initiation, no factor was significantly associated with LTFU.

**Conclusion:**

The factors associated with NFU (being young, not having a treatment supporter, and being diagnosed at hospitals) reflect a vulnerable and potentially highly mobile population. Additional interventions are needed to support and retain this group at ART initiation on Option B+.

## INTRODUCTION

In 2013, the World Health Organization (WHO) updated the guidelines for the prevention of mother-to-child transmission of HIV (PMTCT), recommending that all HIV-infected pregnant, postpartum and breast-feeding women initiate lifelong antiretroviral treatment (ART) as soon as they are diagnosed, irrespective of their CD4 count or WHO clinical staging.^1^ This policy, known as Option B+, was adopted in Tanzania in 2013; implementation began in 2014, and national rollout was achieved by December 2014. In 2015, the PMTCT national programme reported significant improvements in all indicators compared to the prior year. In 15 (55.6%) of the countyr's 27 regions, the national target of ≥90% ART provision to pregnant women was achieved, and mother-to-child transmission of HIV (MTCT) was reduced from 6% in 2014 to 4.5% in 2015. By 2017, 80% of pregnant women living with HIV in Tanzania were receiving ART, a significant increase from 2010, when only 51% of pregnant women were receiving ART.^2^

Option B+ policy was intended to simplify treatment protocols and promote task shifting and service decentralisation in order to increase PMTCT coverage and accelerate progress towards the elimination of perinatal HIV infections.^3^ However, studies have demonstrated suboptimal retention among women initiating ART under Option B+.^4-10^ In particular, loss to follow-up (LTFU) has been reported immediately after HIV diagnosis, with women being initiated on ART, but never returning to the clinic for follow-up appointments.^10,11^ In routine data from Tanzania, rates of LTFU in PMTCT care among women initiating ART under Option B+ were 26%, 30% and 33% at 3, 6, and 12 months respectively.^2^

Several individual factors are associated with retention in PMTCT care, including older age, higher income, higher education level, psychosocial support (including a treatment supporter), and lower levels of stigma and discrimination.^5,7,12^ Health system factors associated with retention include the accessibility and quality of PMTCT services, the attitudes of health facility staff members and the level of the health facility where ART is initiated.^9,11,13^ The influence of these factors on LTFU may vary across time points in pregnancy and the postpartum period.

With the continued scale-up of Option B+, it is important to understand the nuances of care engagement across the PMTCT cascade. Data regarding when LTFU occurs and what influences it can inform strategies that target women most at need and at the most appropriate time. This study aimed to estimate rates of LTFU among women presenting for PMTCT services in Moshi, Tanzania and to identify factors associated with LTFU. In particular, we evaluated factors associated with having no follow-up (NFU) after initial presentation at PMTCT, as well as with LTFU among the women who returned to care at least once after ART initiation.

## METHODS

### Study Setting

This study was conducted in northern Tanzania in Moshi Municipality, an urban setting with a population of 184,292, of which 51.6% and 48.4% are female and male, respectively.^14^ In 2015, there were 60,283 women of reproductive age in the municipality; adult HIV prevalence was estimated to be 3.8%.^14^ Moshi Municipality has a total of 53 health facilities, of which 29 provide PMTCT services. Thirteen of these PMTCT service providers only conduct HIV testing and referral to care. The remaining 16 facilities (of which 3 are hospitals, 3 are health centres, and 10 are dispensaries) provide ART to pregnant women in accordance with Tanzania's national PMTCT programme. PMTCT services provided by Tanzania's PMTCT programme include routine HIV testing and counselling, antiretroviral (ARV) treatment and prophylaxis for mothers and children, safer delivery practices, counselling and support for safer infant feeding practices, long-term follow-up care for mother and child and family planning. PMTCT services are available at antenatal clinics during and after pregnancy until the child is 24 months of age, after which the mother and child are transferred to routine ART care.

### Study Design and Participants

This was a retrospective cohort study, based on routinely collected clinical data of HIV-infected women who initiated Option B+ between February 2014 and December 2015 at any of the 16 PMTCT facilities in Moshi Municipality. The study observation period was from ART initiation until June 30, 2016; thus, all patients included in the analysis had at least 6 months of follow-up data. Eligible patients were those initiated Option B+ because of pregnancy or breastfeeding, irrespective of their clinical or immunological stage. Exclusion criteria for the study were: ART initiation prior to pregnancy, ART initiation at another facility, or missing clinical records. All patients meeting the eligibility criteria were included in the final sample.

### Data Collection and Variable Definitions

Patient information was extracted by a medical doctor and a trained and experienced data clerk in the health facility from paper-based, routinely maintained clinic records using a standardised medical record extraction tool. Study variables were extracted from multiple data sources in the clinic: PMTCT registers, ANC registers, and the patient HIV clinic card. Specifically, we extracted women‘s age, gestational age at ART initiation, and gravidity from ANC and delivery registers. We obtained the patient's marital status, presence or absence of a treatment supporter, and dates of follow-up and appointment visits from the HIV clinic card and the ART and appointment registers. Baseline demographic characteristics and clinical variables, including age at ART initiation, marital status, gestational age at ART initiation, gravidity, treatment supporter status, level of health facility, and eligibility criteria, were extracted for the time of ART initiation. Other variables, such as dates of follow-up and appointment visits, were abstracted on the last ART visit date for both LTFU patients and those retained in care. For all patients, the last ART visit date was the latest date of ART scheduled or unscheduled visit after ART initiation. Extracted data were then entered into Microsoft Excel and imported for analysis into Stata version 12 (StataCorp, College Station, TX, USA).

Two outcomes were considered in this study. The first outcome was NFU, defined as a patient having no recorded follow-up visit after ART initiation. The second outcome was LTFU, defined as a patient being > 90 days late for their last scheduled appointment without subsequently returning to the clinic. For all patients, the last ART visit date was taken as most recent scheduled or unscheduled ART visit occurring before June 30, 2016.

### Sampling and Sample Size

The sample size was calculated using the formula for Poisson distributed data, which is also appropriate for log-binomial models.^15,16^

$${\text{n}}_{group}=\frac{4}{p{\left(\sqrt{RR}-1\right)}^{2}}$$

*p*=probability of event in the unexposed

RR=minimum relative risk of interest

To adjust for unequal group size of women with and without exposure of interest, we used the following formulas:

$$\begin{array}{l} \begin{array}{c} \text{k}={\text{n}}_{un\,\exp osed}/{\text{n}}_{\exp osed}\\ {\text{n}}_{un\,\exp osed}=0.5{\text{n}}_{group}\left(1+\text{k}\right) \end{array}\\ {\text{n}}_{\exp osed}=0.5{\text{n}}_{group}\left(1+1/\text{k}\right) \end{array}$$

The required sample size was n_*unexposed*_ + n_*exposed*_

The following assumptions were applied to calculate sample size: the probability of NFU is 0.15 in unexposed women and 0.20 in the total cohort, exposures of interest have a prevalence ranging from 25% to 75%, RR=2.5, power=80%, and alpha=0.05. Based on these assumptions, the required sample size was 420. The final sample included 468 newly diagnosed HIV positive pregnant women initiating ART on Option B+.

### Data Analysis

Extracted data were entered into Microsoft Excel and then imported for analysis into Stata version 12. Range checks, frequencies and histograms were used to identify missing data and errors. Decisions on how to categorise variables were made based on previous studies. Descriptive statistics were generated. Continuous variables were summarised using means and standard errors (SD) as well as medians and interquartile ranges. Dichotomous variables were summarised using frequencies and percentages. Chi-square tests were used to evaluate bivariate associations between baseline variables and NFU.

Multivariable analyses were conducted. First, we developed a multivariable log-binomial regression model to identify variables that were associated with NFU. The final multivariable model included, *a priori*, age at ART initiation, treatment supporter, level of health facility, and reason for ART initiation, as well as variables with a *P* value <.05 in bivariate analysis. Adjusted relative risks (aRRs) and 95% confidence intervals (CIs) were estimated to assess the magnitude of associations after adjusting for other variables. Secondly, we conducted a survival analysis to estimate Kaplan-Meier survival functions for time to LTFU in the entire cohort and in the subset of women who returned to the clinic at least once after ART initiation. Patients were followed from ART initiation for up to 12 months. Participants were censored at the date of death, date of transfer out of the clinic, LTFU, or at the end of the observation period. In this analysis, we defined the follow-up time of those who never attended a second visit after initiation as 15 days after ART initiation.

Finally, we developed 2 multivariable Cox proportion hazards regression models to evaluate variables associated with time to LTFU, firstly in the entire cohort and then in the subset of women who returned to the clinic at least once after ART initiation. Before developing the multivariable models, univariate Cox proportion hazards regression analyses were conducted to test associations with individual variables. To test the assumption of proportional hazards, we used the “tvc” and “texp” commands in STATA to assess whether or not a linear deviation from proportional hazards would improve the model fit. These tests confirmed that the assumption of proportional hazards was appropriate. The multivariable Cox regression model that best explains the variation in survival (by using a stepwise selection process) included all of the most significant prognostic factors that had a *P* value ≤.10 in uni-variable Cox Proportion hazards analysis. In the final model, we adjusted for age at ART initiation (below or above 25 years), treatment supporter status (yes or no), level of health facility (hospital, health centre, or dispensary), and reason for ART initiation (pregnancy or breastfeeding), regardless of their significance in the univariate analysis. Gravida and gestational age were not included in the final multivariable model because of the high proportion of missing values. As sociations were estimated using hazard ratios (HR) with 95% CIs and were considered significant at a *P* value <0.05 using a 2-sided test.

### Ethical Considerations

Ethical approval to carry out the study was obtained from the Research and Ethics Committee of the Kilimanjaro Christian Medical University College (*Number: 983*). Confidentiality was maintained with no participants' names being used. The study utilised data that are routinely collected for service delivery, and no personal identifiers were included in the data-set used in statistical analyses; therefore, we did not seek patient consent.

## RESULTS

**FIGURE 1.**
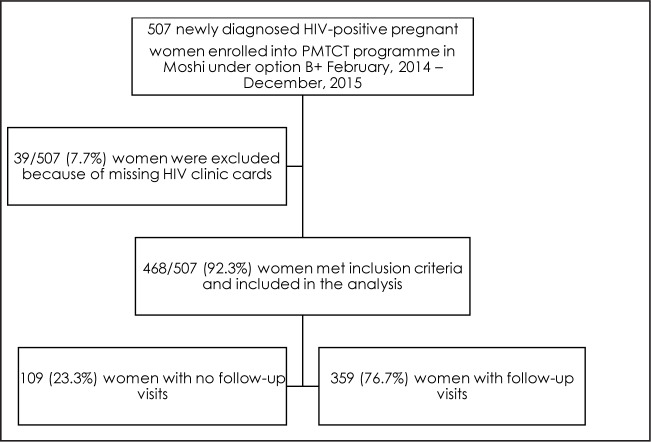
Flow Chart With Total Number of Eligible and Included Women

Between February 2014 and December 2015, 507 newly diagnosed HIV-infected pregnant women were enrolled in PMTCT under Option B+ in Moshi Municipality. After exclusions due to missing data (n=39), data from 468 women were analysed. Of the 468 women, 109 (23.2%) did not return after the ART initiation visit and were classified as NFU, and 359 (76.7%) women had at least 1 follow-up visit after ART initiation (Figure 1).

### Participant Characteristics

Participants ranged in age from 16 to 49 years, with a mean age of 29 years. In total, 346 (73.9%) were aged >25 years and 227 (48.5%) were married/cohabiting. At ART initiation, 284 (60.7%) women were in the first or second trimester of pregnancy, and >50% had a previous pregnancy. In total, 310 (66.2%) women had a treatment supporter, and 443 (94.7 %) women initiated ART during pregnancy, while 25 (5.3%) initiated ART while breastfeeding (Table 1).

**TABLE 1.** Characteristics of Women Initiated on ART Under Option B+ From February 2014 to December 2015 (N=468)

Variable	Total n (%)	Follow-up Status n (%)		X^2^ *P* value
		No Follow-up	With Follow-up	
**Total**	468	109 (23.3)	359 (76.7)	
**Age, years, mean (SD)**	29.29 (6.1)	28.28 (6.8)	29.6 (5.9)	.05^a^
**Age at ART initiation, years**				
<25	122 (26.1)	37 (33.9)	85 (23.7)	.03
≥25	346 (73.9)	72 (66.1)	274 (76.3)	
**Marital status**				
Married	227 (48.5)	54 (49.5)	173 (48.2)	.50
Unmarried	122 (26.1)	33 (30.3)	89 (24.8)	
Missing	119 (25.4)	22 (20.2)	97 (27.0)	
**Gestational age at ART initiation**				
1st or 2nd trimester	284 (60.7)	49 (44.9)	235 (65.5)	.01
3rd trimester or breastfeeding	59 (12.6)	19 (17.5)	40 (11.1)	
Missing	125 (26.7)	41 (37.6)	84 (23.4)	
**Total**	468	109 (23.3)	359 (76.7)	
**Treatment supporter**				
Yes	310 (66.2)	45 (41.3)	265 (73.8)	<.01
No	158 (33.8)	64 (58.7)	94 (26.2)	
**Level of health facility**				
Hospital	143 (30.5)	43 (39.5)	100 (27.9)	.07
Health centre	210 (44.9)	42 (38.5)	168 (46.8)	
Dispensary	115 (24.6)	24 (22.0)	91 (25.3)	
**Reason for ART initiation**				
Pregnancy	443 (94.7)	100 (91.7)	343 (95.5)	.12
Breastfeeding	25 (5.3)	9 (8.3)	16 (4.5)	

a *P* value for Student's t-test

Abbreviations: ART, antiretroviral therapy; SD, standard deviation

### Factors Associated with No Follow-up in ART Care

In the univariate log-binomial analysis, age < 25 years at ART initiation, having no treatment supporter and health facility of ART initiation being a hospital (vs a health centre or dispensary) were significantly associated with NFU. After adjustment, these variables remained independently associated with NFU. Women aged < 25 years had a nearly 2 times higher risk of NFU compared to women aged > 25 years (aRR 1.70; 95% CI, 1.2 to 2.3). Women who had no documented treatment supporter had a nearly 3 times higher risk of NFU compared to those with a documented treatment supporter (aRR 2.86; 95% CI, 2.0 to 3.9). Women who started ART at a hospital had a 50% increased risk of NFU compared to those who started ART at a health centre (aRR 1.50; 95% CI, 1.0 to 2.1) (Table 2).

**TABLE 2.** Factors Associated with No Follow-up on ART Care Among Women Who Initiated ART Under Option B+ (N=468)

	Univariate Analysis		Multivariate Analysis	
Variable	RR (95% CI)	*P* Value^a^	aRR (95% CI)	*P* Value^a^
**Age at ART initiation**				
≥25	1		1	
<25	1.5 (1.0–2.0)	.03	1.7 (1.2–2.3)	<.01
**Marital status**				
Married	1			
Unmarried	1.1 (0.7–1.6)	.50	-	-
**Gestational age at ART initiation**				
1st or 2nd trimester	1			
3rd trimester or breastfeeding	1.9 (1.1–2.9)	<.01	-	-
**Gravidity**				
1st pregnancy	1			
Repeat pregnancy	0.5 (0.2–0.7)	<.01	-	-
**Treatment supporter**				
Yes	1		1	
No	2.8 (2.0–3.8)	<.01	2.9 (2.0–3.9)	<.01
**Level of the health facility**				
Health center	1		1	
Hospital	1.5 (1.0–2.1)	.03	1.5 (1.0–2.1)	.02
Dispensary	1.0 (0.6–1.6)	.85	0.8 (0.5–1.2)	.36
**Reason for ART initiation**				
Pregnancy	1		1	
Breastfeeding	1.6 (0.9–2.7)	.09	1.6 (0.9–2.4)	.05

n (%), Proportion of women with no follow-up in care for each category listed

Abbreviations: aRR, adjusted risk ratio; ART, antiretroviral therapy; CI, confidence interval; RR, risk ratio

### Loss to Follow-up From Care

**FIGURE 2.**
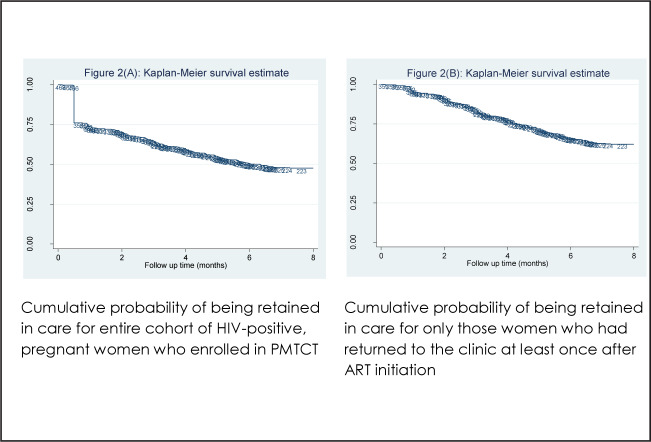
Kaplan–Meier Estimates of LTFU Among Women Starting ART Under Option B+

In total, 468 women were followed for 3,209 person-months (PM). Mean (SD) follow-up time was 8.8 (4.2) months, with a range of 0.1 to 12 months. Overall, 245 (52.3%) women were LTFU during the observation period, including 109 (23.2%) who were lost immediately after ART initiation. The cumulative proportion of women LTFU at 3, 6 and 12 months after ART initiation was 35.4% (95% CI, 31.3 to 39.9), 49.5% (95% CI, 45.1 to 54.1), and 52.3% (95% CI, 47.9 to 56.9), respectively (Figure 2A). The overall incidence rate of LTFU for this cohort, including those who had no follow-up visit after ART initiation, was 7.6 per 100 PM of the observation time (range, 6.7 to 8.6 per 100 PM).

In the analysis of 359 women who returned to the clinic at least once after ART initiation, mean (SD) follow-up time was 8.7 (4.2) months, with a range of 0.1 to 12 months. In total, 136 (37%) of these women were LTFU during the study period. The cumulative proportion of these women LTFU at 3, 6 and 12 months after ART initiation was 15.9% (95% CI, 12.4 to 20.0), 34.2% (95% CI, 29.6 to 39.4) and 37.8% (95% CI, 33.0 to 43.1), respectively (Figure 2B). The overall incidence rate of LTFU for this subgroup of women was 4.3 per 100 PM, with a range of 3.6 to 5.0 per 100 PM.

In assessing LTFU for the entire cohort of 468 participants, age < 25 years at ART initiation (aHR 1.4; 95% CI, 1.0 to 1.8) and having no treatment supporter (aHR 1.8; 95% CI, 1.3 to 2.3) were associated with higher hazards of LTFU. In the subset of women who returned to the clinic at least once after ART initiation, no factor was significantly associated with LTFU (Table 3).

**TABLE 3.** Factors Associated with Loss to Follow-Up Among Women Who Initiated ART Under Option B+ (N=468)

	Univariate Analysis		Multivariate Analysis	
	LTFU^a^ HR (95% CI)	LTFU^b^ HR (95% CI)	LTFU^a^ aHR (95% CI)	LTFU^b^ aHR (95% CI)
**Age at ART initiation, years**				
≥25	1	1	1	1
<25	1.3 (1.0–1.7)^c^	1.2 (0.8–1.8)	1.4 (1.0–1.8)^c^	1.3 (0.8–1.8)
**Marital status**				
Married	1	1		
Unmarried	1.1 (0.7–1.4)	0.9 (0.6–1.4)	-	-
**Gestational age at ART initiation**				
1st or 2nd trimester	1	1		
3rd trimester or breastfeeding	1.4 (0.9–2.0)	1.2 (0.6–1.9)	-	-
**Gravidity**				
1st pregnancy	1	1		
Repeat pregnancy	0.6 (0.4–0.9)	0.8 (0.4–1.2)	-	-
**Treatment supporter**				
Yes	1	1	1	1
No	1.7 (1.3–2.2)^d^	1.1 (0.7–1.6)	1.8 (1.3–2.3)^d^	1.2 (0.8–1.6)
**Health facility level**				
Health centre	1	1	1	1
Hospital	1.1 (0.8–1.5)	0.9 (0.5–1.3)	1.2 (0.8–1.5)	0.9 (0.5–1.3)
Dispensary	0.9 (0.6–1.2)	0.89 (0.5–1.3)	0.8 (0.6–1.1)	0.8 (0.5–1.2)
**Reason for ART initiation**				
Pregnancy	1	1	1	1
Breastfeeding	1.4 (0.8–2.3)	1.2 (0.5–2.5)	1.6 (0.9–2.7)	1.3 (0.6–2.7)

a LTFU for entire cohort of HIV-infected, pregnant women who enrolled in PMTCT between February 2014 and December 2015 (N=468).

b LTFU for only those women who returned to the clinic at least once after ART initiation (N=359).

a HR adjusted for age, treatment support, health facility level and reason for ART initiation

c (<.05),

d (<.001)

Abbreviations: aHR, adjusted hazard ratio; ART, antiretroviral therapy; CI, confidence interval; HR, hazard ratio; LTFU, lost to follow-up

## DISCUSSION

In order to improve the implementation of the Option B+ guidelines for PMTCT, data is needed to understand care engagement across the PMTCT continuum. This study provides insight into PMTCT care engagement in Moshi Municipality, Tanzania, where Option B+ guidelines have been in place since 2014. The study found that almost one-quarter of the women who initiated ART during pregnancy had no record of returning to the clinic after ART initiation. Overall, more than half of the women in the cohort were LTFU during the observation period. Being young, initiating ART at a hospital, and not having a treatment supporter were associated with a greater risk of not returning to the clinic, but not LTFU from subsequent visits.

The study revealed that many women fail to return for follow-up care after initiating ART under Option B+, and also that many women who do return are subsequently LTFU. In this study, we found that at 12 months, LTFU was 37.9%, among women who returned to the clinic after ART initiation. Similar patterns of LTFU have been observed in other African countries implementing Option B+. In Malawi, about half of women who collected ART at initiation never returned for another appointment,^7^ and in Ethiopia, about a quarter of the women who were LTFU received ART only once and never returned.^11^ The high proportion of NFU might suggest that women never started ART, even if they collected ART on the day of initiation, or that they stopped ART after the first dose. However, it is possible that some of these women may attend HIV services in a different location and quite possibly with a different identification number (known as a “silent transfer”).^17^ Women may feel shocked by their HIV diagnosis, and may not be prepared for lifelong ART. Some women may want to receive HIV-related services at another clinic due to perceived stigma and a desire to keep their HIV status secret.^11,17^ The period immediately prior to and after ART initiation is thus an important target for interventions aimed at improving retention in Option B+. Focus should be placed on ways to increase women's readiness for ART and reduce internalised and perceived stigma to improve retention in care.

Our study supports others in suggesting that being younger at ART initiation reduces retention in care.^11,17-19^ Several factors may contribute to poor retention among younger women, including reduced readiness for ART, decreased knowledge of the benefits of PMTCT, as well as stronger feelings of internalised and perceived stigma.^20,21^ Older women may have more social support within partnerships and families which allow them to better manage ART.^7^ Social support, as captured in our study as having a treatment supporter documented in the medical record, was associated with a lower risk of NFU. Having a treatment supporter may be an indication of HIV disclosure, which has been shown to be strongly associated with an individual's ability for sustained engagement in HIV care.^12,22-25^ Disclosing one's HIV status enables one to garner social support and removes the element of secrecy; women who have disclosed their HIV status are more likely to show up to their appointments without fear.^25-28^ The present study relied on routinely collected clinical data, which did not include details about the characteristic of the treatment supporter, including the individual's relationship with the patient and the type of support they provide. Further research should explore the characteristics of treatment supporters that facilitate care engagement.

Our data supports other studies showing that women who initiate ART in tertiary health-care facilities are at increased risk of NFU, compared with women who initiate ART at lower-level facilities.^4^ Poorer retention in higher-level health facilities may be related to unfavourable health system factors such as high patient loads and longer waiting times,^4,11^ or may be linked to the increased mobility of urban populations.^29^ However, it may also be that women are not prepared to receive their ART care at the same location that they had selected for their pregnancy care. Further research is needed to determine if women who have NFU following ART initiation on Option B+ continue their care in other locations, and to examine their subsequent retention in these locations.

Our study has several limitations. The routine data extracted from the health facilities were often missing information on factors including marital status, gestational age at ART initiation, gravidity and parity. Other potentially important factors reported in other studies are not captured in the routine data (eg, HIV stigma and barriers to health-care access). Baseline characteristics were used as fixed variables to assess associations with the NFU and LTFU; however, some of these factors may change over time. Lastly, we were not able to trace women who may have moved to other facilities, and therefore, our analysis may have overestimated NFU and LTFU. In clinical settings such as those of the study area, health-care workers often do not actively trace patients who are LTFU, and transfers to other facilities are not recorded unless patients specifically request a transfer letter. Thus patients who self-transfer to another facility may be misclassified as NFU or LTFU in routine data.

## CONCLUSION

Our analysis of routine clinic data from Option B+ programmes in Northern Tanzania revealed a high proportion of HIV-infected pregnant women who initiated ART but never returned for follow-up care or an ART refill, a trend which may undermine the success of Option B+. Younger women, those initiating ART in hospitals and those with no reported treatment supporter were at increased risk of early dropout. These women may have transferred their HIV care to other facilities or may have disengaged completely. Further prospective quantitative and qualitative studies should be carried out to understand the reasons for and factors associated with early LTFU from HIV care in the context of Option B+ in Tanzania. Future reporting on PMTCT programme outcomes at the national level should disaggregate data by age and other variables to better assess early LTFU.
